# Protein Export via the Type III Secretion System of the Bacterial Flagellum

**DOI:** 10.3390/biom11020186

**Published:** 2021-01-29

**Authors:** Manuel Halte, Marc Erhardt

**Affiliations:** Institute for Biology–Bacterial Physiology, Humboldt-Universität zu Berlin, Philippstr. 13, 10115 Berlin, Germany; manuel.halte@hu-berlin.de

**Keywords:** bacterial flagellum, flagellar assembly, type III protein export, ATPase, proton motive force, secretion model

## Abstract

The bacterial flagellum and the related virulence-associated injectisome system of pathogenic bacteria utilize a type III secretion system (T3SS) to export substrate proteins across the inner membrane in a proton motive force-dependent manner. The T3SS is composed of an export gate (FliPQR/FlhA/FlhB) located in the flagellar basal body and an associated soluble ATPase complex in the cytoplasm (FliHIJ). Here, we summarise recent insights into the structure, assembly and protein secretion mechanisms of the T3SS with a focus on energy transduction and protein transport across the cytoplasmic membrane.

## 1. Introduction

Flagella are complex rotary nanomachines embedded in the cell envelope of many bacteria. In addition to functions in adhering to surfaces, flagella allow bacteria to move in their environment towards nutrients or to escape harmful molecules. They are present in both Gram-negative and Gram-positive bacteria, and are evolutionary related to the injectisome device, which various Gram-negative bacterial species use to inject effectors into eukaryotic target cells [1]. Both the flagellum and injectisome are complex nanomachines and made of around 20 different proteins, ranging from a copy number of very few to several thousand [2]. Structurally, the flagellum can be divided into three main parts: (i) a basal body embedded in the cell envelope; (ii) a flexible linking structure, the hook; and (iii) a long external filament, which functions as the propeller.

**Figure 1:**
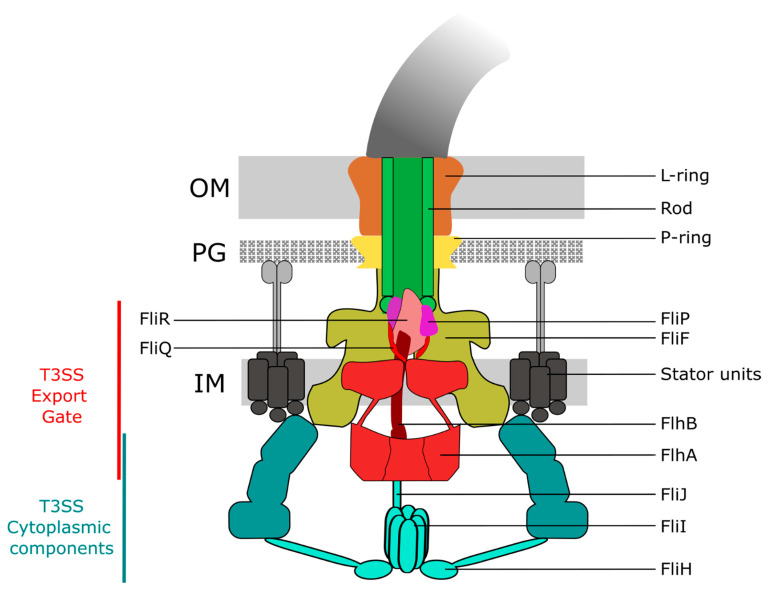
Schematic of the flagellar basal body complex. The basal body consists of the stator units, the periplasmic rod, the P- and L rings embedded in the peptidoglycan (PG) and outer membrane (OM), respectively, the MS ring in the inner membrane (IM), and the T3SS. The T3SS is composed of cytoplasmic components (the C ring and the ATPase complex consisting of FliI, FliH and FliJ), and the export gate (consisting of FliPQR, FlhA and FlhB) located at the base of the basal body within the MS ring.

The basal body is composed of the rod (made of FliE, FlgB, FlgC, FlgF, and FlgG) and several protein rings: the MS ring (made of FliF) in the inner membrane (IM) and the C ring (made of FliG, FliM and FliN) in the cytoplasm, the periplasmic P ring located in the peptidoglycan (PG) layer (made of FlgI), and the L ring (made of FlgH) in the outer membrane (OM) [3]. The assembly and function of both the flagellum and injectisome relies on a conserved protein export apparatus located at the base of the basal body. The so-called type III secretion system (T3SS) found in the flagellum (flagellar T3SS; fT3SS) and injectisome (virulence-associated T3SS; vT3SS) is comprised of five conserved core export gate proteins, FlhA/SctV, FlhB/SctU, FliP/SctR, FliQ/SctS, and FliR/SctT, and is associated to three soluble proteins, FliH/SctL, FliI/SctN, and FliJ/SctO, respectively, for the fT3SS and vT3SS, forming the export apparatus (Figure 1) [4,5,6]. The T3SS is involved in substrate protein selection, i.e., docking of substrate proteins (and their cognate secretion chaperones) to the export apparatus, the subsequent unfolding of substrates and proton motive force-driven translocation across the inner membrane, and forms the central secretion pore through which the substrates are secreted. As the diameter of the secretion channel is only 2 nm, secreted proteins are likely in an (at least partially) unfolded state [7]. Additionally, the T3SS can discriminate between different substrate classes. Only upon completion of the hook–basal–body (HBB) complex, the T3SS switches substrate specificity from early type substrates (e.g., components of the rod or the hook subunits) to late-type substrates (e.g., flagellin or the filament cap), which enables a mechanism to prevent premature secretion of the many thousand flagellin subunits before the assembly of the hook is completed [8]. This switch in substrate specificity involves an interaction between a molecular ruler protein, FliK, and the export gate protein FlhB [9,10,11] in case of the flagellum, while a homologous ruler protein (SctU/SctP) is implicated in substrate selectivity switching of the injectisome [12].

Furthermore, protein secretion via the T3SS is remarkably fast. The T3SS is able to secrete several thousand amino acid per second [13], in comparison to the general secretion system (Sec system) that only secretes a few dozen amino acids per second [14,15]. In recent years, a model has emerged where primarily the proton motive force (PMF), i.e., the charge and proton gradient across the inner membrane, provides energy to drive protein translocation across the inner membrane via the T3SS [16]. The soluble ATPase complex made of FliHIJ is part of the export apparatus and thought to facilitate docking and unfolding of substrates [17]. Accordingly, the secretion of T3SS substrate proteins can be described as a three-step process: (i) docking of substrate proteins, which might be associated with their chaperones, to the export apparatus; (ii) unfolding of substrate proteins; and (iii) PMF-dependent injection of substrate proteins into the secretion channel. The secreted substrate proteins subsequently travel (presumably in an α-helical or partially unfolded conformation [18]) through the channel inside the flagellum to the tip, where they self-assemble.

This chapter highlights the protein components that make up the core T3SS of the flagellum, discusses potential mechanisms underlying the substrate energization processes, summarizes the various models that have been proposed to understand the secretion process and assembly of flagellin subunits into the growing filament and compares the protein export mechanism of the flagellar T3SS to the protein secretion mechanisms used by other bacterial secretion systems (Box 1).

## 2. Export Apparatus Structure and Assembly

**Figure 2:**
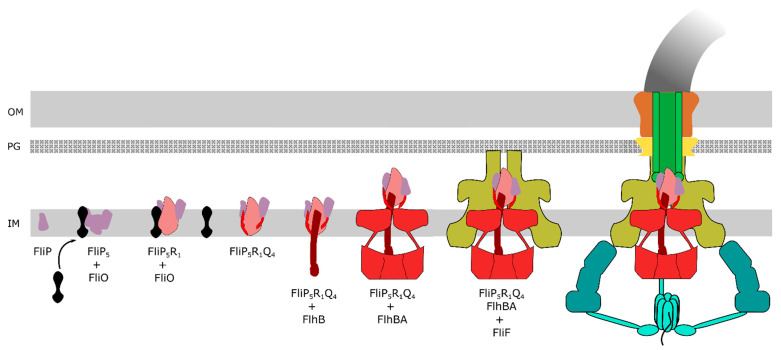
Step-wise export apparatus assembly model. Formation of the FliP_5_ complex in the IM is presumably promoted by the integral-membrane chaperone FliO. FliO further facilitates the formation of a stable FliP_5_R_1_ complex. Once the FliP_5_R_1_ complex is formed, FliO is thought to dissociate from the complex. The FliP_5_R_1_ complex then constitutes the nucleus for the assembly of the subsequent subunits of the export gate (FliQ_4_, FlhB and FlhA_9_). Finally, the MS ring made of FliF forms around the core export apparatus FliP_5_FliQ_4_FliR_1_FlhB_1_FlhA_9_ in the IM followed by the assembly of the C ring (made of FliG/M/N) and recruitment of the ATPase complex.

The core export gate of the flagellar T3SS is located in the centre of the basal body and formed by FliPQR, FlhA and FlhB. Associated at the cytoplasmic face is the ATPase complex constituted of FliHIJ. FliPQR form a helical assembly with 5:4:1 stoichiometry (FliP_5_Q_4_R_1_) embedded in the core of the basal body and located above the inner membrane (IM) [19]. A small bitopic membrane protein, FliO, was previously thought to be part of the export apparatus. However, it has recently been shown that FliO is not part of the assembled basal body complex and that FliO has no active role in the export process, but functions as a chaperone for productive assembly of the FliP-FliR complex [20] (Figure 2). It is interesting to note that, although FliPQR homologs are strongly conserved in the vT3SS, no homologs of FliO have been found thus far, which suggests a specific role for assembly of the fT3SS [21]. It has been postulated that FliO promotes stable FliP sub-assemblies in the IM, before forming a complex with FliR. Once this complex is formed, FliO would dissociate, and the complex would interact with FliQ to form the FliP_5_Q_4_R_1_ helical assembly [19] before interacting with FlhB and finally FlhA. FlhB consists of an N-terminal, transmembrane (TM) domain (FlhB_TM_) formed by four α-helices, and a C-terminal cytoplasmic domain (FlhB_C_). FlhB_C_ is responsible for the switching from early type substrates (e.g., the rod and hook subunits) to late-type substrates (e.g., the anti-sigma factor FlgM, the hook-filament junctions, the filament tip and flagellin). This switching requires a proteolytic autocleavage of FlhB_C_ between Asn269 and Pro270 at a highly conserved NPTH motif splitting FlhB_C_ into FlhB_CN_ and FlhB_CC_, in addition to an interaction with the molecular ruler FliK [12,22]. In the autocleavage deficient mutant FlhB(N269A), the T3SS is deficient for the secretion of late-type substrates [23]. Under normal conditions of flagellar assembly, FliK is secreted intermittently throughout the hook construction period: once the hook reached a length of ~55 nm, the N-terminus of FliK remains in the channel, thereby slowing-down its secretion. Presumably, this leaves the FliK C-terminal domain enough time to interact with FlhBc, inducing a conformational change in the export apparatus that results in the switch in substrate specificity [11]. Following the switch in secretion specificity, the anti-sigma factor FlgM is recognized as a late secretion substrate and secreted. Secretion of FlgM releases σ^28^ and allows for σ^28^-dependent gene expressions from class III promoters, including *fliC* encoding for the flagellin FliC [8,24,25]. A recent study on the structure of the core flagellar export apparatus validated that FlhB is part of the export gate [26]. Interestingly, FlhB associates to the core secretion pore FliPQR by forming a loop (FlhB_L_), containing the most conserved residues of FlhB. The four helices in the FlhB_TM_ domain form two distinct hairpins that wrap around the cytoplasmic face of the FliPQR complex, inserting hydrophobic residues in the cavities between FliQs subunits. Kuhlen et al., initially hypothesised that FlhB_L_ is involved in maintaining a closed export gate, but the FliPQR structure from a *flhB* deletion mutant is present in a closed conformation [26]. The wrapping of FlhB_L_ might be involved in the opening of the export gate, either by moving away of the entrance with a hinge motion similar to a lid, or by staying in contact with the opening FliQs subunits and extending its structure in a similar way to a sphincter. Deletions of residues in FlhB_L_ led to loss of motility, and the extensive interactions on the cytoplasmic face and on the surface of the FliPQR complex with FlhB demonstrates that FlhB is an integral component of the export gate complex directly involved in substrate protein secretion [26]. FlhA is a central component of the PMF-driven flagellar protein export machine and hypothesised to function as the proton/protein antiporter. FlhA can be separated into two regions: a hydrophobic N-terminal transmembrane (TM) region with eight predicted α-helical transmembrane domains (FlhA_TM_) that interact with the MS ring [27], and a hydrophilic C-terminal cytoplasmic region (FlhA_C_) that interacts with FliHIJ and FlhB, and with the substrate-chaperone complex (hook-filament junction protein FlgK/FlgN, filament capping protein FliD/FliT and flagellin FliC/FliS) [28,29,30,31]. The FlhA_C_ region consists of four domains: D1 (residues 362 to 434 and residues 484 to 503), D2 (residues 435 to 483), D3 (residues 504 to 583), D4 (residues 584 to 682), and a flexible linker termed FlhA_L_ (residues 328 to 361). The FlhA_C_ regions form a nonameric, cytoplasmic ring beneath the export gate through D1-D3 and D3-D3 interactions [32], and is involved in the recognition of substrates, the binding of the substrate-chaperone complex and the actual secretion process [30,33].

Upon assembly of the core secretion pore, the switch protein FlhB and the nonameric FlhA ring, the MS ring assembles in the inner membrane around the flagellar export gate. The MS ring functions as a scaffold and is formed by 34 subunits of FliF [34]. The FliG/M/N proteins (respectively 26–34, 34 and around 100 copies [35]) form the cytoplasmic C ring, which interacts tightly with the MS ring. Although it was proposed previously that 26 copies of FliG are present in the C ring [35], the stoichiometry of FliF and FliG are likely matched, suggesting that the FliG part of the C ring consists of 34 subunits [34]. The C ring primarily functions as the rotor the flagellum, which through interactions with the stator units formed by MotA_5_B_2_ drives rotation of the flagellum. The FliG subunits of the C ring interact with the rotating stator units (a pentamer of MotA that has been proposed to rotate around a dimer of MotB using energy derived from the ion gradient across the inner membrane), and enables bidirectional rotation of the flagellum by changing its conformation upon binding of the phosphorylated response regulator CheY during chemotaxis [36,37,38]. Additionally, the C ring plays a role as affinity site for substrates [39].

## 3. Structure and Function of the ATPase

As mentioned above, a soluble ATPase complex formed by FliHIJ is located at the cytoplasmic interface of the flagellar basal body. The ATPase FliI is found in two different complexes, (i) in the FliI_6_FliH_12_FliJ complex at the base of the export apparatus, where FliI forms a hexameric ring, and (ii) in the freely diffusing FliH_2_FliI hetero-trimer [40,41,42,43,44]. The FliI_6_FliH_12_FliJ complex is associated to the C ring through interactions between FliH and FliN/FlhA, and FliJ might play a role in the energy coupling mechanism for flagellar protein export by interacting with FlhA [28,45]. The structure of the complex SctV/SctO of *Chlamydia pneumoniae* (vT3SS homologs of FlhA/FliJ, respectively) indicates that interaction of SctO to the C-terminal region of SctV alters the binding site for substrate-chaperone complex and changes the conformation of SctVc. A potential rotation of FliJ (discussed in more details below) might then release the substrate-chaperone complex, allowing subsequent secretion of the substrate protein [46]. However, the exact role of the ATPase complex for protein secretion via the flagellar export apparatus remains poorly understood. Under physiological conditions, the role of the ATPase complex appears to be facilitating efficient secretion of substrate proteins via the T3SS. Interestingly, however, the FliHIJ ATPase complex has been shown to be dispensable for filament formation under certain conditions, such as overproduction of flagellar substrates or when the cell’s available PMF is increased [16,47,48]. In support, several mutations in FliI that abolish or substantially reduce its ATPase activity are still able to assemble flagellar filaments, suggesting that any process energized by ATP hydrolysis is uncoupled from the actual protein secretion mechanism [49].

The ATPase FliI is a member of the Walker-type ATPase family. It shares similar structural characteristics with the α/β subunits of the F_O_F_1_ ATP synthase [50]. In contrast to the F_O_F_1_ ATP synthase where the α and β subunits forms a hetero-hexamer, FliI forms a homo-hexamer. Hexamer formation is needed for the enzyme to exert its full ATPase activity. FliJ binds to the FliI_6_ ring and functions to stabilise the formation of the hexameric FliI ring, which then resembles the F1-α_3_β_3_γ complex [28,51]. FliH is divided into three regions: an N-terminal region FliH_N_, a central region FliH_M_ and the C-terminal region FliH_C_. Only the first 10 amino acids of FliHc are critical for the export of substrates through the secretion channel by binding with the C ring through FliN-FliH interactions [45]. FliH promotes the interaction of FliI_6_FliJ with the C ring by interacting through its C-terminal region with FliI and N-terminal region with FliM-FliN [45]. Photocrosslinking experiments have additionally revealed an interaction between FliH and FlhA_C_, but not with the other proteins of the basal body. It is assumed that the interaction of FliH_N_ and FlhA_C_ anchors the FliI_6_FliH_12_FliJ ATPase complex to the export apparatus during the protein secretion process [52]. The second, soluble FliI complex (FliH_2_FliI) has been shown to inhibit formation of the hexameric FliI_6_ ring, and to bind to late export substrates in complex with their chaperones [40,41,43,44,53,54]. In this function, the FliH_2_FliI complex is thought to act as a dynamic carrier to deliver FliJ and the late export substrate-chaperone complexes to the docking platform of the T3SS export gate formed by FlhA_C_ [43,55]. The FliH_2_FliI carrier binds to the hook-filament junction protein FlgK/FlgN and filament capping protein FliD/FliT substrate-chaperone complexes, but not to FliC/FliS [53,56,57]. It has been proposed that FliH_2_FliI may contribute to efficient interactions of the FlgK/FlgN and FliD/FliT substrate chaperone complexes, promoting an efficient assembly of the hook/cap structures before the assembly of the filament [58]. The interaction of the FliH C-terminal region with the FliI N-terminal region inhibits its ATPase activity, indicating that the N-terminal of FliI might be involved in the regulation of the ATPase activity. This inhibition might prevent consumption of ATP before the export of substrates. The presence of another factor (FliJ) is then required to start the activity of FliI [40].

It has been proposed that FliJ might also act partially as a rotor: the V_o_V_1_ and F_O_F_1_ ATPases use a rotational mechanism to hydrolyse ATP, and since the FliI_6_FliJ complex and the F1-α_3_β_3_γ complex are evolutionary related and share structural similarities, it appears possible that the two systems use a similar mechanism for distinct functions [59]. Accordingly, a rotational mechanism has recently been proposed to explain how the ATPase FliI_6_FliJ might assist the unfolding of substrates before their export into the channel. For this ATPase rotation mechanism, FliJ would function as the rotor and bind in the central cavity of the hexameric FliI ring, which itself is anchored to the C ring via FliH interactions and thereby would function as the stator. Further, each FliI monomer is predicted to bind an ATP molecule and harbour a FliJ binding site. When FliJ is bound to one FliI monomer, it cannot rotate. After hydrolysis of ATP in one of the FliI monomers, FliJ is released from its confinement and can freely rotate temporarily. The motion is governed by two parameters: (i) ATP hydrolysis rate and (ii) lifetime of the ADP-bound state. As a folded substrate protein arrives at the export gate, ATP hydrolysis would then induce rotation of the FliJ shaft, and the rotating FliJ interacts with the folded substrate (or substrate-chaperone complex), providing sufficient energy to help overcome the energy barrier and unfold the substrate protein (or strip-off the chaperone) before PMF-dependent injection into the secretion channel. Such a model is in agreement with previous observation that a certain level of protein export still occurs in the absence of the ATPase complex, suggesting that the complex is merely helping in the substrate protein export process [60]. Cryo-EM structure determination of the ATPase SctN (vT3SS FliI homolog) and its central stalk SctO (vT3SS FliJ homolog) of enteropathogenic *Escherichia coli* identified the presence of hydrophobic residues that contribute to the interaction between SctN and SctO, and which might facilitate rotation of SctO in a similar way to the F_1_-ATPases. In such a mechanism, the SctO/FliJ stalk could stabilize the ATPase complex to facilitate binding of the chaperone. A transition from an ATP- to ADP-bound state of the ATPase might then cause the dissociation of the chaperone from its substrate protein and the ATPase complex, thereby facilitating subsequent substrate secretion [61].

It is now clear that the ATPase FliI of the flagellar T3SS only has a facilitating role in substrate protein secretion via the T3SS. The ATPase is not required for substrate translocation per se, as a Δ*fliHI* double mutant is still able to produce filaments with low probability. In contrast to the dispensability of the ATPase complex for the construction of flagella in vivo [16,47,51], however, the analysis of flagellar T3SS protein transport in an in vitro model using inverted cytoplasmic membrane vesicles (IMVs) showed confusingly that the secretion of early and late substrates was dependent on the presence of the cytoplasmic FliH_2_FliI complex, while abolishing the PMF did not affect secretion [17]. A potential explanation of these contradicting observations might be that the concentration of the FliH_2_FliI complex used in the in vitro experiments was kept constant, and higher than under in vivo conditions. Such conditions might allow substrates transport into the IMVs via the fT3SS in the absence of the PMF. Accordingly, further investigations are required to determine the molecular mechanism how the cytoplasmic ATPase complex contributes to export substrate targeting, unfolding and opening of the transmembrane export gate.

## 4. Translocation of Substrate Proteins

### 4.1. The Role of the PMF and ATPase

How translocation of substrate proteins via the T3SS is coupled to ATP hydrolysis by the soluble cytoplasmic ATPase and the PMF across the cytoplasmic membrane remains poorly understood. The PMF is made up of two components, the electric potential (Δ_Ψ_) and the transmembrane proton gradient (ΔpH) and results from the translocation of protons by the electron transport chain across the IM. Importantly, the PMF is involved in several major biological processes including ATP synthesis by the F_O_F_1_-ATP synthase and for various transport processes. The PMF also plays a major role in energizing substrate protein secretion via T3SS. Disruption of the PMF in *Yersinia peptis* impaired secretion of Yop effector proteins [62]. Further, addition of an uncoupler that disrupts the PMF such as carbonyl cyanide *m*-chlorophenylhydrazone, abolished flagellar protein secretion in *Salmonella enterica* [16]. Interestingly, flagellation of mutant of the FliI ATPase complex can be restored to nearly wild-type levels by overexpression of late secretion substrates (e.g., using a mutant of the anti-σ^28^ factor FlgM) or by increasing the available PMF (e.g., by deleting the F_O_F_1_-ATP synthase as a major consumer of the PMF during ATP regeneration via electron transport phosphorylation) [48]. Similar observations were made in the vT3SS of *Pseudomonas aeruginosa*, where a cytoplasmic regulator and a cytoplasmic component control the access of effectors and the overall secretion by modulating the conversion efficiency of the PMF for protein export, adding yet another level of regulation [63].

While the overall importance of the PMF in energizing substrate protein secretion via T3SS is now rather clear, the underlying molecular mechanism of how the export apparatus couples the PMF to substrate protein translation remains unknown. Both components of the PMF (ΔpH and Δ_Ψ_) might have different roles during substrate protein secretion, as it has been observed that only Δ_Ψ_ is necessary to promote protein translocation in wild-type cells, while both components are necessary in a mutant that allows flagellar substrate secretion in the absence of the T3SS ATPase FliI. This Δ*fliHI flhB*(P28T) bypass mutant secretes the early type substrates FliK and FlgD in equivalent amount as wild-type cells, adding evidence to the suggestion that the function of the ATPase FliI is not essential for the actual protein secretion process. Disruption of the PMF in the wild-type and the Δ*fliHI flhB*(P28T) bypass mutant abolished protein secretion and filament formation. An increase in ΔpH improved the secretion of the Δ*fliHI flhB*(P28T) bypass mutant, and use of deuterium oxide D_2_O (heavy water isotope impacting the rate of proton translocation) led to a high decrease in FlgD secretion level only in the Δ*fliHI flhB*(P28T) bypass mutant, while the secretion levels of wild-type cells remained unchanged. Accordingly, the rate of proton translocation via the T3SS appears to limit protein export in the absence of FliHI [16,51].

Many mutations in the FlhA/FlhB components of the export gate have been identified that impact the formation of the flagellar filament and might help us to understand the mechanism of PMF-driven protein export. As mentioned above, the FlhB(P28T) mutation was shown to significantly improve the formation of flagella in absence of FliHI, but how this mutation actually bypasses the loss of the ATPase has remained obscure [47]. The recently solved structure of the export gate FliP_5_Q_4_R_1_FlhB_1_ revealed that the location of the FlhB N-terminal region (containing residue P28) at the cytoplasmic entrance of the gate might affect closure and opening of the gate. In support, in case of the vT3SS, a SctU_F28pBpa_ mutant was shown to interact with SctS (vT3SS homolog of FliQ) by in vivo photocrosslinking. As the FlhB_TM_ domain wraps around the export gate components FliPQR, the conformational change necessary for the opening of the export gate might happen from pulling forces imparted on helix 4 of the FlhB_TM_ domain, linked to the other helixes through conserved buried charged residues, including FlhB D208. This force might be initiated in FlhA, the only component of the export apparatus demonstrated to have multiple conformations [64]. The FlhB D208A mutation in FlhB_TM_ also disrupted motility, but was rescued by an overexpression of FlhA, highlighting the importance of a charged residues link between the two proteins [26].

### 4.2. The Role of FlhA

As mentioned previously, FlhA can be separated into two regions: a hydrophobic N-terminal transmembrane (TM) region with eight predicted α-helical transmembrane domains (FlhA_TM_), and a hydrophilic C-terminal cytoplasmic region (FlhA_C_) consisting of four domains: D1 (residues 362 to 434 and residues 484 to 503), D2 (residues 435 to 483), D3 (residues 504 to 583), D4 (residues 584 to 682). A flexible linker termed FlhA_L_ connects the TM8 of the N-terminal region with the D1 domain of the C-terminal cytoplasmic region. D1-D3 and D3-D3 interactions within FlhA_C_ subunits form a nonameric, cytoplasmic ring beneath the export gate [32].

As FlhA is a central component of PMF-driven flagellar protein export via the T3SS, several mutagenesis studies of conserved charged residues have been performed in an attempt to identify potential proton binding sites. This section discusses the structure and function of FlhA in T3SS protein export and highlights the mutations that affect substrate protein secretion, by affecting the interaction with substrate-chaperone complexes or by disrupting proton binding and the proton-driven conformational changes in FlhA.

Interactions of FlhA with FliHIJ and FlhB/FliPQR play an important role for the protein secretion process. FlhA_L_ and a hydrophobic dimple located between D1 and D2 interact with FliJ and substrate-chaperone complexes, promoted by FliI and FliH. The interaction of FlhA with the ATPase complex might allow the export gate to efficiently utilize the PMF to facilitate flagellar protein export [28]. As the D2 domain is directly involved in the translocation of substrates, it has been proposed that FliH and FliI promote the interactions of FlhA to FliJ and substrate-chaperone complex to ensure an efficient energy coupling mechanism [55]. Based on mutational analysis, the D1 domain of FlhA might be involved in substrate entry into the secretion channel [65]. The flexible linker FlhA_L_ is further required for FlhA_C_ oligomerisation as alanine substitutions in the D1 domain (interacting with FlhA_L_) and in FlhA_L_ inhibit the formation of the FlhA_C_ ring. These mutations reduce the efficiency of filament assembly after completion of the hook, as well as the binding affinity of FlgN, the chaperone of FlgKL [29]. Triple alanine substitution in the D1 domain resulted in a polyhook phenotype, indicating that the mutations potentially affected hook-length control. Other alanine substitutions mutants in FlhA_L_ domain still assembled a complete hook structure, although hook length was not tightly regulated anymore, and secretion of FlgM (and accordingly of FlgK and FliC) was reduced (while early substrates secretion e.g., of FlgE/FlgD was not impaired). As hooks/basal bodies were still produced at the wild-type level, this suggests that these residues are involved in the interaction with late-type substrates and that the interactions of FlhA_L_ with its neighbouring subunit may induce conformational changes in the FlhA_C_ ring structure to initiate the export of filament-type proteins (e.g., FlgM/FlgK) once the hook has been completed [29]. The V404M mutation in the D1 domain of FlhA facilitates binding of FliI in absence of FliH to the export apparatus. FlhA_C_(G368C) is a temperature-sensitive mutant which displays significantly reduced flagellar protein export at elevated temperatures but not at the permissive temperature of 30 °C [66,67,68]. Thermal stability experiments showed that this mutation affects denaturation of the C-terminal domain of FlhA_C_, revealing the importance of FlhA conformational rearrangements for productive protein secretion [64,65]. Hara et al. identified the non-motile D208A mutation and suggested that this charged residue might be directly involved in the PMF-driven protein export. However, a more extensive mutagenesis study revealed that proton-binding at this position is non-essential for flagellar protein export, as suppressor mutations elsewhere in FlhA were able to rescue the motility defect of D208 mutants. In addition, significant transport activity was measured in D208 mutants using a more sensitive assay based on export of a hook protein fusion to beta-lactamase (FlgE-Bla) [69,70].

**Figure 3:**
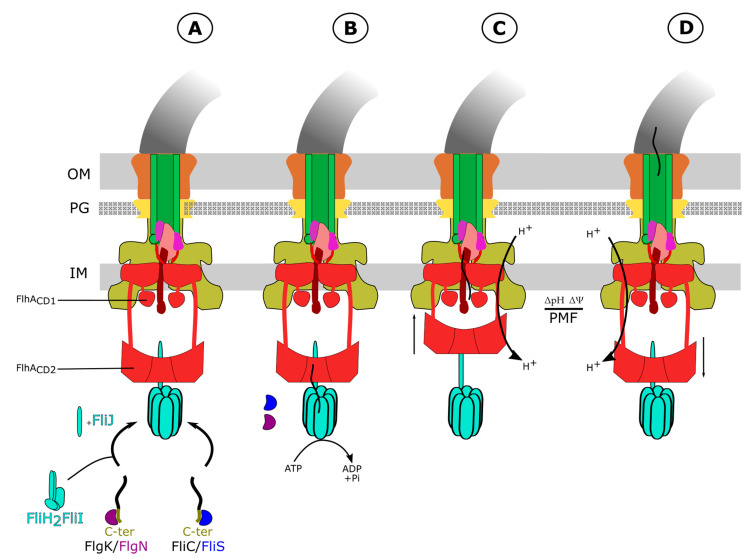
Putative model of the fT3SS secretion process. (**A**) Delivery of chaperones/substrates complexes to the FliH_12_FliI_6_FliJ ATPase complex, followed by (**B**) removal of the chaperone/unfolding of the substrates mediated by ATP hydrolysis of the ATPase complex FliH_12_FliI_6_FliJ and delivery to FlhA. (**C**) Proton-driven cyclical movements of the FlhA_CD2_ ring cause an opening of the export gate and mediate cycles of secretion substrate binding and release into the secretion channel. (**D**) Diffusion of secreted substrates inside the secretion channel to the tip of the flagellar structure.

In the same study, three other charged residues (R147, R154 and D158) located in a small cytoplasmic loop between TM4 and TM5 (FlhA_CD1_) were identified as necessary for FlhA function and potential proton binding sites [70]. This highly conserved cytoplasmic loop between TM4 and TM5 coordinates the secretion of early secretion substrates during hook assembly by interacting with the FlhA_C_ domain. This suggests that substrate entry in the channel is regulated by this loop. A protonation-mimicking mutation in this loop (FlhA(D158N)) induces a large conformational change of the FlhA_C_ domain, and it therefore has been proposed that during the secretion process the cytoplasmic FlhA_C_ ring gets close to the loop between TM4 and TM5 through this proton-driven conformational change [70]. This protonation mimicking mutation triggers a global conformational change that also affects the large cytoplasmic domain of FlhA (FlhA_C_, also termed FlhA_CD2_ in order to discriminate it from the cytoplasmic loop between TM4 and TM5 termed FlhA_CD1_), which interacts with secretion substrates. These data led to a model of how proton-driven conformational cycling of FlhA_CD1_ might drive substrate protein secretion. In this model, the nine copies of FlhA form two cytoplasmic rings; the FlhA_CD1_ ring positioned close to the membrane and the larger FlhA_CD2_ ring more distal to the export gate. It is presumed that FlhA_CD2_ can cycle between its more distal position from the membrane and a position more proximal to the membrane, where it is held in place through interactions with FlhA_CD1_. The model further proposes that proton-driven conformational changes in FlhA_CD1_ modulate these interactions to drive the cyclical movements of the large FlhA_CD2_ ring between the positions proximal and distal to the membrane (Figure 3). The proton-driven cyclical movements of the FlhA_CD2_ ring are presumably linked to cycles of secretion substrate binding and release into the secretion channel [70].

However, several crucial steps of the flagellar protein secretion process remain unclear. First, while the relation between FlhA and FlhB evidently is vital for an efficient PMF-dependent secretion of substrate proteins via the fT3SS, how exactly the T3SS distinguishes between early and late-type substrates is unknown. The proton-driven cyclical movements of FlhA_CD1_ and FlhA_CD2_ might impact FlhB, causing an opening of the export gate. Recent mutational analysis suggests that a structural rearrangement of the FlhA_C_ ring is promoted by interaction between FlhB_C_ and FlhA_C_. A FlhA(A489E) suppressor mutant partially rescues the impaired late-type substrates secretion of FlhB(P270A) (a slow-cleaving FlhB autocleavage mutant, which forms polyhooks) and shortens the length of the polyhooks. These observations suggest that the FlhA(A489E) mutation assists the FlhB(P270A) mutant in switching to late-substrate secretion mode. Interestingly, the FlhA(A489E) mutation is located in the binding site for substrate-chaperone complexes and reduces the binding affinity for these complexes. Accordingly, it appears reasonable to conclude the FlhA(A489E) mutation mimics the conformation of the chaperone-bound FlhA_C_ ring also in presence of the FlhB(P270A) mutation. In a *fliK*-null mutant, the FlhA(A489E) FlhB(P270A) double mutant is able to secrete early type substrates (e.g., FlgE) at a wild-type level, while late-type substrates secretion (e.g., FliC) is abolished. This result highlights the crucial role of the molecular ruler FliK for some structural rearrangement of the FlhA_C_ ring that enables late substrate-chaperone docking. Accordingly, these mutational results support a model, where the molecular ruler FliK interacts with FlhB_C_ as the hook reaches its final length of ~55nm. This interaction induces a conformational change in FlhB required for cleavage of FlhB_C_. The cleaved FlhB_C_ in turn interacts with FlhA_C_ and causes a conformational change that promotes interaction of the FlhA_C_ ring with the substrate-chaperone complexes, thereby enabling the secretion of late-type substrates [71].

Further, the role of the ATPase complex in the actual substrate secretion process remains poorly understood. It has been suggested that cycles of ATP hydrolysis may induce conformational changes in the export gate that result in opening of the gate, as the FlhA_C_ ring is known to interact with FliJ and to be positioned between the export gate and the ATPase complex [26]. Based on cryo-electron tomogram imaging of basal body complexes in situ, the distance between the export gate and ATPase is large, and it is thus unclear how the ATPase complex would be able to mediate the gate opening. Kuhlen et al. proposed that the gate-opening conformational changes would presumably be mediated via interactions of the FliJ stalk of the ATPase complex with the cytoplasmic FlhA_CD2_ ring [26]. Further, as outlined above, low levels of flagellar protein secretion is possible also in the absence of the ATPase complex and therefore, a spontaneous opening of the export gate complex must be possible. In such a model, where conformational changes in FlhB are driven via FliI-FliJ-FlhA interactions, the activity of the ATPase complex might only be required for the initial gate opening. It would appear reasonable to assume that, once opened, the export gate then remains open as long as substrate proteins are transported.

SctV, the vT3SS homolog of FlhA, also forms a nonameric ring and binds substrate-chaperone complexes before substrate secretion. The substrate-chaperone binding site of SctV is, however, not conserved in FlhA, as in place of the hydrophobic dimple found in FlhA, the SctV substrate-chaperone binding site is made of highly conserved charged residues glutamine and arginine located at the interface between the D3 and D4 domains [33]. As the fT3SS needs to switch substrate specificity from early to late-type substrate secretion, the vT3SS requires two specificity switches; the first from early to intermediate-type substrates, and the second from intermediate- to late-type substrates. The first switch might be similar to the switch of the fT3SS, as the second one requires the presence of a gatekeeper protein, SctW, which is absent in the fT3SS. This gatekeeper interacts with the C-terminal and membrane domains of SctV (the vT3SS homolog of FlhA), and this binding decreases the binding affinity of the chaperone/effector complexes to SctV, allowing secretion of translocators but preventing an early secretion of effectors before contact with the eukaryotic host cell. Substrates of the translocator class might also contain an N-terminal signal sequence that is recognized by the gatekeeper protein. Upon host cell contact, SctW dissociates from SctV, the binding affinity of effectors for SctV increases, which subsequently allows secretion of the effector class of vT3SS substrates [72].

## 5. Model for High-Speed Secretion of Flagellin

In the previous chapters, we discussed the organisation of the flagellar T3SS and how substrate proteins might be translocated across the inner membrane in an ATP- and PMF-dependent manner. However, the flagellum grows by polymerization of its building blocks at the distal end. Flagellin is pumped into the secretion channel, travels through the secretion channel and then polymerises on top of the completed hook at the distal end of the flagellum with the help of the filament cap FliD [73,74]. Around 20,000 subunits of flagellin assemble to form the flagellar filament, which can grow up to a 20 µm in length [25,75]. Accordingly, once translocated into the two nm narrow secretion channel, the many thousand building blocks of the flagellum must travel distances ranging from several dozen nanometer (e.g., in case of hook subunits) up to several micrometer (in case of flagellin subunits). This raised the question if transport beyond the inner membrane relied on additional energy sources. Several models have been proposed on how the bacteria might be able to transport thousands of building blocks over such long distances.

Initial observations of flagellar filament growth were made using electron microscopy-based measurements of filament lengths in vitro and in vivo. In vivo experiments showed an exponential decrease of the filament growth rate with increasing filament length [13], while in vitro measurements revealed a constant growth rate [76]. However, the flagellum grows by incorporating flagellin subunits at the tip of the filament and the in vitro measurements do not consider the transport of flagellin molecules inside the narrow secretion channel from the base to the tip. Accordingly, the authors concluded that the exponential decay was caused by a decrease in translocation efficiency [13]. Such a mechanism is supported by several theoretical and computational analyses: Keener presented a biophysical model for the growth of flagellar filaments where the monomers are translocated into the channel by the ATPase and then diffuse until reaching the tip where they assemble. Such a model resulted in similar qualitative results to the measurements made for the exponential decay. The quantitative differences with the experimental measurements were hypothesised by the fact that the movement of the monomer substrates is not driven solely by diffusion [77]. Using molecular dynamics simulation, Tanner et al. proposed a mathematical model of the translocation-elongation process which describes all of the properties included in the elongation process: the friction of the flagellin during interactions with the channel, the flagellin density and the flagellin translocation rate. This theoretical model was consistent with the exponential measurement of Iino et al., and the authors concluded that the flagellum growth rate decreases exponentially with length because of protein compression and friction between translocating flagellin and the flagellar channel [78].

An alternative, electrostatic model has been proposed to be responsible for secretion of effector proteins through the needle of the injectisome. In this model, secretion substrate proteins with charged residues would travel through the channel by electrostatic repulsion mediated through negative charged residues present on the inside of the channel wall. Since the negative charges would be present throughout the length of the needle, the electrostatic repulsion would enable the transport of the substrate proteins from the base of the channel to the tip. In this model, the insertion of the positive charged substrates inside the channel would be energized by the PMF-driven T3SS, which would push the substrate proteins into the needle channel [79]. However, this model requires the presence of negative electrostatic charges inside the secretion channel, and more recent study on the structure of the injectisome needle revealed that the inner linings of the channel wall is mainly neutral [80]. The flagellar channel is also mostly hydrophilic, probably to minimize hydrophobic interaction with the unfolded substrates that would hinder the diffusion to the distal end of the filament. Interestingly, among the lining residues, at least two amino acids are positively charged and/or polar for the flagellin FliC of *Salmonella*, and this number is generally up to four amino acids as shown by alignment of flagellin homologs. Most of the amino acids in the channel are polar non-charged, and although one lining residue of the flagellar filament channel in *Salmonella* is negatively charged, the presence of two other lining positively charged residues seem to indicate either a positive or neutral global charge inside the channel, tending to the idea that electrostatic repulsion could not propel substrate proteins through the channel [7,81].

The current model for the filament growth mechanism of the bacterial flagellum is based on diffusive motion of single flagellin molecules inside the filament channel. Evidence supporting such a mechanism was first published in 2012 when Turner et al. developed a clever technique to estimate the elongation rate of individual flagellar filaments. They used a flagellin variant harbouring a surface-exposed cysteine replacement mutation, which allowed to visualize filament fragments using maleimide-coupled fluorophores. After shearing of the filament, Turner et al. labelled the filament using maleimide dyes of two different colours and observed that the filament was able to re-grow, and that the length of the second fragment was independent of the length of the first fragment [82]. A length-independent growth mechanism was puzzling, however, and not consistent with the previous observations of an exponential decay of filament growth. Stern et al. predicted that PMF-dependent injection of partially folded, α_-_helical flagellin subunits followed by single-file diffusion inside the channel, would account for linear filament growth provided that the diffusion coefficient of flagellin molecules is sufficiently large [83].

An alternative model to explain the length-independent filament growth and resulting constant rate of flagellin transport was based on the observation that secretion substrates are captured by the C-terminal domain of FlhB through head-to-tail linkage of terminal helices [84]. This led to a model where a chain of head-to-tail linked unfolded flagellin subunits would span the complete length of the secretion channel from the export gate to the tip of the filament. Crystallization of the most distal flagellin subunit at the tip would exert a force to pull the next subunit from the export gate, maintaining a constant rate of flagellin subunit transport to the tip of the flagellum. While elegant, this model of inter-subunit chain formation is not compatible with the simultaneous secretion of non-chaining substrates such as the anti-σ factor FlgM or excess hook-associated proteins (FlgK, FlgL, FliD), which are known to be continuously secreted during the assembly process [85], but do not interact with FliC [86]. The chain model can also not explain the secretion of non-flagellar proteins fused to an N-terminal T3SS signal peptide (e.g., FlgM) and subsequently exported via the flagellar protein secretion system [87]. Finally, premature termination of translation is occurring frequently [88] and presumably result in a sub-population of C-terminal truncated secretion substrates, which would be unable to form inter-subunit chains. This was confirmed by co-expression of truncated flagellin unable to form inter-subunit chains and to assemble into the filament, which did not affect the filament elongation kinetics [89].

In 2017, Renault et al. provided further evidence in support of a model of flagellar filament growth that is dependent on PMF-driven injection of flagellin subunits into the empty secretion channel and one-dimensional diffusion inside the channel from the base to the distal tip [89]. Here, the site-specific labelling of flagellin subunits containing a surface-exposed cysteine residue using maleimide-coupled fluorochromes was optimized by exchanging dyes multiple times in situ during normal bacterial growth. The high-resolution multiple labelling approach revealed a length-dependent filament growth with an elongation speed that gradually decreased from ~100 nm⋅min^−1^ to ~20 nm⋅min^−1^ for 8 µm long filaments, which translates to an initial flagellin secretion rate of ~1700 amino acids per second. In an earlier study that investigated the growth rate of the filament, Iino used p-fluorophenylalanine (pFPA), which incorporates into the growing filament and changes the filament curvature. The incorporation of pFPA results in a curly filament that can be distinguished from the usual filament structure and enabled Iino to estimate the growth of filament fragments after addition of pFPA. Using this technique, Iino observed that the rate of elongation decreases exponentially with the increase in flagellar filament length, and attributed this to a decrease in the efficiency of flagellin transportation through the secretion channel. However, the decreased transport efficiency was not fully understood at that time, as the initial fast growth rate caused by the injection was not integrated in Iino equations [13]. The more recent injection-diffusion model considers the injection of the substrates into the channel, the diffusion coefficient of the monomer inside the channel, the length of the monomer and the increment in length of the flagellum. For more precise, quantitative measurements of the filament elongation rate, Renault et al. further employed a strain where the master regulator FlhDC expression is dependent of an inducible promoter. This enabled Renault et al. to control the timing of filament initiation and growth. Using the maleimide approach described above, the authors could determine the growth rate of several different filament segments over time. They found that the growth rate of a new, distal filament fragments was inversely proportional to the initial basal-fragment length. This approach also allowed to exclude broken filaments from the analysis [89]. In support of the injection-diffusion model, a length-dependent growth of the flagellar filament was also observed by Zhao et al. in *Vibrio alginolyticus* using real-time fluorescent labelling of the flagellar sheath, as well as in *E. coli* [90,91]. Here, real time fluorescence microscopy of filament growth confirmed that flagellar growth rate was inversely proportional to the length and further revealed that an insufficient cytoplasmic flagellin supply results in intermittent pauses during filament elongation in *E. coli* [90].

## 6. Conclusions

The T3SS of Gram-negative bacteria is a protein secretion machine that primarily uses a PMF-driven mechanism to translocate its substrate proteins across the inner membrane into a narrow secretion channel, and is essential for the assembly of complex nanomachines such as the flagellum and injectisome. The associated ATPase complex might have a facilitating role during the secretion process by activating the PMF-driven export gate or facilitating secretion substrate docking and unfolding. Among all of the secretion systems developed by bacteria, the T3SS displays striking features. It facilitates the secretion of substrate proteins from the cytoplasm at a remarkably high speed of several thousand amino acids per second in a one step process through the IM and OM, using both ATPase hydrolysis and the PMF. Although some principle aspects of the protein secretion process via the T3SS are now well-established, numerous questions remain on the underlying molecular mechanisms of the actual secretion and the energisation processes. In particular, it remains to be elucidated how the T3SS is able to transport substrate proteins at such a high rate while preventing the leakage of small molecules. How different classes of substrates are recognized and what constitutes the switch in secretion substrate specificity remain equally elusive. Considering its dispensability at least for secretion of flagellar proteins, the role of the cytoplasmic ATPase complex and its potential contribution to substrate targeting, unfolding and chaperone release continues to be a big mystery. Finally, the holy grail of the T3SS secretion mechanism will be a molecular understanding of how the PMF is actually coupled to the protein secretion process.

Box 1 **Energy requirements of other bacterial secretion systems.**

Bacteria have evolved different ways to secrete proteins into the external environment and/or into mammalian or plant hosts cells. Proteins are secreted into the periplasm or inserted in the inner membrane by the general secretion system (Sec) and the Twin-arginine translocation (Tat), which secrete unfolded and folded (co-factor containing) proteins, respectively. The Sec system is found in all domains of life, while the Tat system is found in bacteria and archaea. Protein translocation through the Sec system is powered by the ATPase SecA, which couples ATP hydrolysis to substrate protein insertion into the protein conducting channel formed by the translocase SecYEG. The PMF is required for a high substrate secretion rate, but it is currently unknown which component of the PMF is necessary. Usage of the PMF was proposed to favour the outward flow of the substrate polypeptide in a Brownian ratchet-type mechanism. It is still under debate how ATP hydrolysis and the PMF enable substrate protein translocation, however. Two main models have been proposed to explain it: the Brownian ratchet model for the ATP-driven reaction, potentially aided by the PMF, and a “push-and-slide” model, involving diffusion and powerstroke movements of the components driven by the ATPase SecA. There also seems to be a critical role of the phospholipids of the membrane. Notably, cardiolipin a specialized phospholipid, which is necessary for the stability of the complex, and for both ATP and PMF-driven protein translocation activity [92,93], indicating that an essential lipid-protein interface exists for the secretion process. Membranes lacking cardiolipin are unable anymore of PMF stimulated translocation, suggesting a direct role of cardiolipin on the diffusion of the protons. Contrary to the Sec system, substrate protein translocation through the Tat system (formed by TatA, TatB and TatC) is exclusively driven by the PMF. In vivo studies have shown that the Δ_Ψ_ component alone is sufficient to energize protein translocation. The assembly of TatA is also promoted by the PMF, but the mechanism how the PMF contributes to the assembly of the Tat system remains unknown. Currently, two different models exist to explain substrate protein translocation by the Tat system; the first is based on pore formation in the membrane, and the second on membrane weakening by TatA complexes, where short TM domains would locally reduce the membrane thickness after binding of the cargo proteins [94].

Among other protein secretion systems of Gram-negative bacteria, the T2SS and T5SS utilize a two-step protein secretion mechanism, as their substrates are first translocated into the periplasm in a Sec/Tat- and Sec-dependent manner, respectively, before getting translocated outside of the bacterial cell. The T2SS (e.g., responsible for secretion of the cholera toxin of *Vibrio cholerae* or the heat-labile enterotoxin of enterotoxigenic *Escherichia coli*) can be separated in four subassemblies: the OM complex, the IM platform, the secretion ATPase in the cytoplasm, and the pseudopilus in the periplasm. The secretion ATPase interacts with the IM platform at its cytoplasmic interface and the OM complex translocates the substrates through the outer membrane. The energy for the translocation is provided by the ATPase in the cytoplasm, as ATPase mutants with no activity are unable to secrete effectors. ATP hydrolysis by the ATPase induces the formation of the pseudopilus, that in turn pushes exoprotein substrates through the OM complex channel by alternating extension and retraction similar to a piston, and comparable to what can be observed for type 4 pilus assembly systems (T4PS) [95,96]. The high similarity between T4PS and T2SS, as well as to archaeal flagella, suggest an early common origin, potentially from archetypal structure that evolved in specialized T4PS and T2SS pseudopilus [97]. T5SS are autotransporters that can be classified into monomeric and trimeric autotransporters, and two-partner secretion systems (TPSS). Several types of T5SS exist, reviewed in Leo et al. [98]. Contrary to the others Gram-negative protein secretion systems, the energy requirement for the translocation of T5SS is not provided by ATP hydrolysis or the PMF, but by protein folding. It is presumed that the folding of the C-terminal domain of the secreted substrate protein in the extracellular space acts as a Brownian ratchet to move passively across the OM [99].

The T1SS and T4SS are one-step protein secretion systems that use energy derived from ATP hydrolysis to drive substrate protein transport from the cytoplasm through the IM and OM. T4SS form pili that are able to extend and retract, and are used for the transfer of DNA from a donor to a recipient cell by a process called conjugation, or to secrete proteins to the outside of the cell envelope. The cycles of pilus extension/retraction are powered by ATP hydrolysis of a dedicated ATPase, and pilin subunits are reinserted in the membrane during the retraction cycles. The pilin subunits are processed in the IM before incorporation into the growing pilus and assemble into the pilus at the base of the T4SS. In the case of the T4SS from F-like plasmids, it is now known that F-like pili are not composed solely of pilin, but also of phospholipids from the inner membrane (mainly phosphatidylglycerol 32:1 and phosphatidylglycerol 34:1, which are the two most common phosphatidylglycerol species found in the bacterial cell membrane) in a stoichiometric manner. The presence of these lipids inside of the F-like pili structure would then help the transfer of the ssDNA through the T4SS by making the channel moderately electronegative (without phosphatidylglycerol, the pilus lumen is overwhelmingly electropositive). The presence of phospholipids in the structure might also lower the energetic barrier for the extraction or re-insertion of pilus subunits from, or into, the inner membrane, thereby facilitating pilus extension or retraction, respectively. The lowered energy barrier may also facilitate pilus insertion into the recipient cell membrane for efficient cargo delivery [100]. Interestingly, the pilin subunit TraA is integrated in the inner membrane and extracted from it with a phosphatidylglycerol using an ATP and PMF dependent process, independent of the Sec pathway. Depletion of the PMF causes an inhibition of pilin processing, which in turn prevents pilus assembly and the conjugation process [101].

## Data Availability

Not applicable.
